# Improving the lesion appearance on FLAIR images synthetized from quantitative MRI: a fast, hybrid approach

**DOI:** 10.1007/s10334-024-01198-z

**Published:** 2024-08-24

**Authors:** Fei Xu, Stefano Mandija, Jordi P. D. Kleinloog, Hongyan Liu, Oscar van der Heide, Anja G. van der Kolk, Jan Willem Dankbaar, Cornelis A. T. van den Berg, Alessandro Sbrizzi

**Affiliations:** 1 Computational Imaging Group for MR Diagnostics & Therapy, Center for Image Sciences, University Medical Center Utrecht, Utrecht, The Netherlands; 2 Department of Radiotherapy, University Medical Center Utrecht, Utrecht, The Netherlands; 3 Department of Medical Imaging, Radboud University Medical Center, Nijmegen, The Netherlands; 4 Department of Radiology, University Medical Center Utrecht, Utrecht, The Netherlands

**Keywords:** Quantitative MRI, Synthetic MRI, FLAIR, Brain MRI

## Abstract

**Objective:**

The image quality of synthetized FLAIR (fluid attenuated inversion recovery) images is generally inferior to its conventional counterpart, especially regarding the lesion contrast mismatch. This work aimed to improve the lesion appearance through a hybrid methodology.

**Materials and methods:**

We combined a full brain 5-min MR-STAT acquisition followed by FLAIR synthetization step with an ultra-under sampled conventional FLAIR sequence and performed the retrospective and prospective analysis of the proposed method on the patient datasets and a healthy volunteer.

**Results:**

All performance metrics of the proposed hybrid FLAIR images on patient datasets were significantly higher than those of the physics-based FLAIR images (*p* < 0.005), and comparable to those of conventional FLAIR images. The small difference between prospective and retrospective analysis on a healthy volunteer demonstrated the validity of the retrospective analysis of the hybrid method as presented for the patient datasets.

**Discussion:**

The proposed hybrid FLAIR achieved an improved lesion appearance in the clinical cases with neurological diseases compared to the physics-based FLAIR images, Future prospective work on patient data will address the validation of the method from a diagnostic perspective by radiological inspection of the new images over a larger patient cohort.

**Supplementary Information:**

The online version contains supplementary material available at 10.1007/s10334-024-01198-z.

## Introduction

Magnetic resonance imaging provides high sensitivity for evaluating intracranial pathologies due to its excellent soft tissue contrast [1]. However, the scan time of conventional brain magnetic resonance imaging (MRI) is rather long due to the sequential acquisition of multiple sequences with different contrast weightings. Compared to conventional MRI, synthetic MRI produces multiple contrast-weighted images from a single quantitative MRI (qMRI) acquisition, providing valuable information for radiological evaluations in a reduced MR examination time [2–5]. Most synthetization techniques also provide quantitative information about the physical properties of the tissue to potentially assist in lesions characterization tasks [3].

Synthetic MRI allows to generate conventional contrast weighted images (such as fluid-attenuated inversion recovery (FLAIR)) by adjusting the imaging parameters offline without the need for additional scanning. Tanenbaum et al. (2017) and Kleinloog et al. (2023) reported that synthetic images have, in general, similar image quality compared to that of conventional MRI [6, 7]. However, for FLAIR images, it was found that the image quality is inferior to their conventional counterpart: brain lesions often appeared hypointense on synthetic FLAIR images, whereas they appeared hyperintense on conventional FLAIR images. Also, synthetic FLAIR image generation based on other qMRI techniques, such as Multiple-Dynamic Multiple-Echo (MDME), Magnetic Resonance Fingerprinting (MRF) -based synthetic MRI, suffers from the contrast related issues with FLAIR images [8]. This contrast mismatch arises from the certain unmodeled physical effects, including but not limited to partial volume, magnetization transfer, diffusion, susceptibility [3, 9] and it indicates the shortcomings of physics-based synthetization approaches.

Recently, several studies introduced different deep-learning methods to improve the quality of synthetic FLAIR images [10–14]; for instance, Chatterjee et al. employed a hybrid method to correct synthetic FLAIR contrasts by combining keyhole strategies and deep learning-based image reconstruction with a mild acceleration factor of 3 [14]. However, these data-driven approaches rely on homogeneous datasets for training and face challenges when dealing with type of sequences or sets of sequence parameters which are not part of the training data. Consequently, separate networks need to be trained for each MR protocol [13]. For this reason, we do not consider data-driven approaches in this work.

In this study, we present an approach to address the contrast mismatch in the lesion region within synthetic FLAIR images. Based on a full brain 5-min Magnetic Resonance Spin TomogrAphy in Time-domain (MR-STAT) acquisition [15], which delivers quantitative *T*1, *T*2, and proton density maps, we enhance the FLAIR synthetization by additionally acquiring an ultra-undersampled conventional FLAIR sequence with an acceleration factor of 8 which retains tissue contrast information and circumvents the need for overly complex physics-based models. We focus on improving the lesion appearance; for this reason, we reduce the high frequency artifacts derived from the cross-contrast combination step by using a soft keyhole approach and a normalization method [16–18]. No generic, population-based data-driven deep learning prior is needed.

This technique was validated on forty neurological patients and shows that FLAIR lesion contrasts can be accurately synthesized at the cost of a 13% increase in acquisition time for a full brain quantitative protocol.

## Materials and methods

### Quantitative imaging

Quantitative MR imaging was performed by using a multi 2D axial MR-STAT sequence to map multiple parameters (e.g. *T*1, *T*2, and PD) simultaneously from a five-minute full-brain scan [7, 15]. The MR-STAT sequence is a spoiled gradient echo acquisition method that uses a non-selective inversion pulse followed by flip angles varying between 0° and 90° in concomitance with a Cartesian-encoded sampling.

For the MR-STAT, the problem can be written in a matrix format with a factorization of the time-domain signal [19]:

1 $$\underset{\alpha }{\text{min}}\frac{1}{2}{\left\Vert D-\sum_{i=1}^{{N}_{y}}{C}_{i}^{p}UY({\alpha }_{i}){C}^{r}({\alpha }_{i})\right\Vert }_{F}^{2}$$

where $D\in {\mathbb{C}}^{{N}_{Tr}\times {N}_{Read}}$ is the measured signal, ${N}_{y}$ is the number of voxel in phase encoding (*y*) direction, ${C}_{i}^{p}\in {\mathbb{C}}{ }^{{N}_{Tr}\times {N}_{Tr}}$ is the diagonal phase encoding matrix for the $i$-th line of voxel in *y* direction, $U\in {\mathbb{C}}^{{N}_{Tr}\times {N}_{Eig}}$ is the SVD-based compression matrix for echo time signal, $Y({\alpha }_{i})\in {\mathbb{C}}^{{N}_{Eig}\times {N}_{x}}$ is the compressed echo time signal for the $i$-th line of voxel, ${C}^{r}({\alpha }_{i})\in {\mathbb{C}}^{{N}_{x}\times {N}_{Read}}$ is the readout encoding matrix for the $i$-th line of voxel.

The reconstruction of $\alpha$ (i.e. *T*_1_, *T*_2_, and proton density maps) was performed according to the alternating direction method of multipliers (ADMM) method present by Liu et al. [19].

### Physics-based FLAIR

A physics-based signal model that is fully determined by the quantified tissue parameters and sequence parameters was used for the synthetic FLAIR images [7, 20]:

2 $$S_{P} = PD.\frac{{1 - 2e^{{\left( { - \frac{TI}{{T_{1} }}} \right)}} + e^{{\left( { - \frac{TR}{{T_{1} }}} \right)}} }}{{1 + e^{{\left( { - \frac{TR}{{T_{1} }}} \right)}} \cos \theta }}.e^{{\left( { - \frac{TE}{{T_{2} }}} \right)}}$$

where $PD$ is proton density, $TI$ is inversion time, $TR$ is repetition time, $TE$ is echo time, ${T}_{1}$ is longitudinal relaxation time, ${T}_{2}$ is transverse relaxation time, $\theta$ is flip angle.

The process to generate images in this way is referred to as “physics-based” synthetization. Previously published studies demonstrated that physics-based synthetic FLAIR images show a sub-optimal lesion appearance compared to conventional FLAIR images due to certain un-modeled physical effects, including but not limited to: magnetization transfer (MT), partial volume, diffusion, and susceptibility [3, 9].

One dominant effect contributing to the contrast mismatch is related to MT. The MT effect arises from the interaction between protons in a “free pool” (bulk water) and a “bound pool” (protons bound in proteins and other large macromolecules). Although these pools share the same central resonance frequency, the bound pool has a much broader spectral width, allowing it to be selectively saturated by off-resonance power deposition [21]. In TSE images, strong MT effects are evident due to long echo trains with multiple refocusing pulses, which result in off-resonance power deposition in slices adjacent to the excited slice [22, 23]. In contrast, MT effects are expected to have a lower influence on gradient echo based MR-STAT acquisitions, which include only a single refocusing pulse.

While it is possible to extend the quantitative imaging protocol to map these additional effects, the scan time can become impractical, and the physical modeling can be challenging. As a result, in the following section we will introduce an alternative synthetization method.

### Hybrid framework

**Fig. 1:**
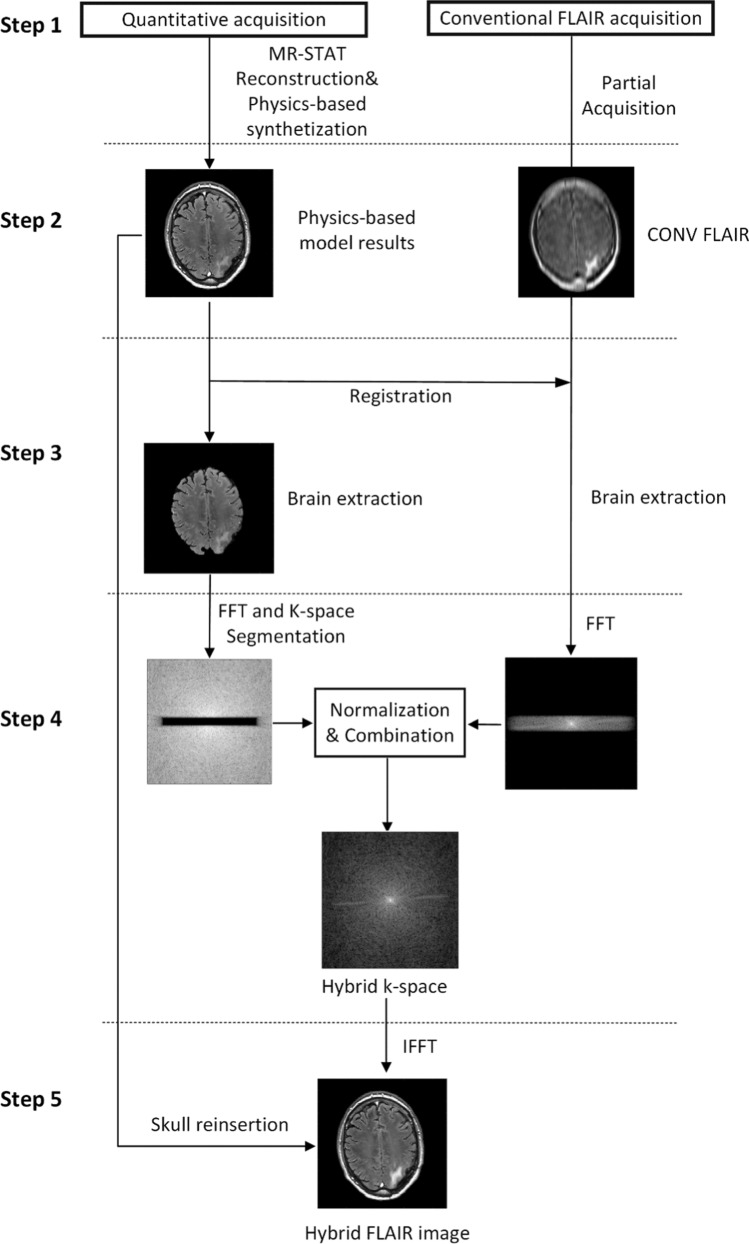
Flowchart of the proposed hybrid FLAIR synthetization method

The proposed “hybrid” acquisition and reconstruction procedure consists of the following five steps (see Fig. 1):

Step 1: full brain MR-STAT acquisition (five minutes) and undersampled acquisition (same field of view (FOV) and resolution of the MR-STAT sequence) of conventional FLAIR data. The MR-STAT reconstruction is represented by Eq. (1).

Step 2: preliminary physics-based synthetic FLAIR images are reconstructed with MR-STAT quantitative parameter maps (*T*1, *T*2 and proton density) as input. See Eq. (2).

Step 3: registration and brain-only extraction. We apply a registration step by using a rigid transformation model with the MATLAB inbuilt ‘‘imregister’’ function to align the conventional low resolution FLAIR images to the physics-based synthetic FLAIR images. Subsequently, we apply a BET (Brain Extraction Tool) from the FSL toolbox to remove the skull area [24]. The fractional intensity threshold is set to 0.5. This step eliminates artifacts caused by high-intensity signals in extra-cranial and non-brain tissues. The removed skull area will be reinserted in step 5.

Step 4: K-space normalization (see Eq. (3)) and combination (see Eq. (4)) are performed, where the central region (contrast information) of the physics-based FLAIR k-space is replaced by the ultra-undersampled conventional FLAIR k-space.

Step 5: the hybrid FLAIR image is finally obtained by taking the Fourier transform of the combined k-space and reinserting the skull region from the physics-based FLAIR image.

#### Image normalization

A mismatch between the image intensities in the physics-based FLAIR and conventional FLAIR may affect the hybrid FLAIR quality. Here, k-space data for each FLAIR image was normalized. The normalization scaling factor is defined by the following equation:

3 $$N_{factor} = \frac{{RMS\left({\mathbf{\,{F}}\left({S_{C,R}} \right)\,.\,w\left({y_{R}} \right)}\right)}}{{RMS\left({\mathbf{\,{F}}\left({S_{P}}\right)\,.\,w\left({y_{R}}\right)}\right)}},$$

where RMS is the root mean square, $\mathbf{\,{F}}$ is Fourier transform and $w({y}_{R})$ is a smoothing Tukey window function for soft thresholoding, ${y}_{R}$ is the phase encoding lines with an undersampling factor of *R*, ${S}_{C,R}$ is the undersampled conventional FLAIR image with an undersamling factor of *R,* and ${S}_{P}$ is the physics-based FLAIR image.

#### K-space combination with Tukey window function

A hybrid FLAIR image can be reconstructed by the following equation:

4 $$H_{f} = {\mathbf{\,{F}}}^{ - 1} \left( {{\mathbf{\,{F}}}\left( {S_{C,R} } \right)\,w\left( {y_{R} } \right) + N_{factor} \cdot\,{\mathbf{ \,{F}}}\left( {S_{P} } \right)\cdot\left( {1 - w\left( {y_{R} } \right)} \right)} \right)$$

where ${\mathbf{\,{F}}}^{-1}$ is inverse Fourie transform.

The Tukey window, also known as the *cosine-tapered window*, is defined as follows [25]:

5 $$w\left( {y_{R} } \right) = \left\{ {\begin{array}{*{20}l} {\frac{1}{2}\left\{ {1 + \cos \left( {\frac{2\pi }{r}\left[ {y_{R} - \frac{r}{2}} \right]} \right)} \right\}, 0 \le y_{R} \le \frac{r}{2}} \\ {1, \frac{r}{2} \le y_{R} \le 1 - \frac{r}{2}} \\ {\frac{1}{2}\left\{ {1 + \cos \left( {\frac{2\pi }{r}\left[ {y_{R} - 1 + \frac{r}{2}} \right]} \right)} \right\}, 1 - \frac{r}{2} \le y_{R} \le 1} \\ \end{array} } \right.$$

where $r$ is the ratio of cosine-tapered section length to the entire window length with $0\le r\le 1$.

Algorithmic parameters were empirically set to optimize the reconstructed hybrid FLAIR based on visual inspection. In this section, we outline this step.

The smoothing Tukey window serves the purpose of minimizing the artifacts caused by residual discontinuities in the frequency distribution when the high-frequency and low-frequency components are combined. To illustrate this point, we conducted a comparison using one patient dataset, where we applied both a rectangular-shaped window, a Hann-shaped window and the window we used in this study ($r$ = 0.25), see Figure S1. When using a rectangular shape window, we observed truncation artifacts, while the use of a Hann shape window resulted in heavy blurring and less improvement in lesion appearance. Therefore, the Tukey window represents a trade-off between these two kinds of artifacts and therefore was chosen in this study.

For heavily undersampled acquisitions of conventional FLAIR, we explored accelerating factors across a wide range (*R* = 16, 8, 4, 2). The data from the conventional FLAIR and the physics-based FLAIR was merged using a Tukey window function (Eq. 4). To determine the optimal accelerating factor of the conventional acquisition, hybrid FLAIR images were reconstructed by applying these four undersampling factors to fully sampled conventional FLAIR from one tumor patient. The comparison among physics-based synthetization, hybrid synthetization with different accelerating factors and fully sampled conventional FLAIR images of a tumor patient is depicted in Figure S2. In terms of SSIM, the values for *R* = 16 and *R* = 8 are very close. However, visually, the lesion indicated by the arrow is better highlighted for *R* = 8 compared to R = 16. Similar observations across other datasets have led us to select R = 8 for this study.

### Study participants and design

#### Image acquisition and processing

All study participants underwent quantitative 2D axial MR-STAT imaging and conventional fully sampled 2D axial FLAIR imaging on a 3 T Ingenia MRI scanner with a 15-channel receiver head coil (Philips, Best). Sequence parameters are shown in Table 1. For the patient scans, fully sampled conventional FLAIR data were acquired and retrospectively under-sampled. To demonstrate the validity of this retrospective acquisition analysis, one additional healthy volunteer was scanned, and both fully sampled and partially sampled FLAIR data was prospectively acquired. The scan time for the partially sampled 2D FLAIR includes the preparation and calibration time (20 s) and the duration for acquiring the k-space data (20 s). The proposed hybrid approach was applied as described above.

**Table 1:** Acquisition parameters of the MR-STAT and conventional FLAIR sequence

Imaging Parameters	MR-STAT Spoiled-GRE	FLAIR TSE (Fully sampled)	FLAIR TSE (12.5% sampling)
Scan time	300 s	200 s	40 s
FOV/resolution	224 × 224 × 133.5 mm^3^/1 × 1 × 3 mm^3^		
Gap	1.5 mm		
Slices	30		
TR (msec)	8.9	10,000	
TI (msec)	–	2800	
TE (msec)	4.7	120	
Flip angle	Variable	90	

GRE = Gradient Echo; TSE = Turbo Spin Echo;

### Patient datasets

Patient datasets were already acquired in a recently completed clinical study [7], where whole brain MR-STAT and conventional FLAIR images were acquired in 40 patients with 4 different neurological diseases, namely: brain tumor (*n* = 11), epilepsy (*n* = 10), multiple sclerosis (MS, *n* = 10) and stroke (*n* = 9). The study was approved by the local Ethic Review Board (NL69544.041.19, METC 19/282) and written informed consent was obtained before inclusion of the study participants.

### Retrospective analysis on patient datasets

To assess the efficacy of improving lesion appearance with the proposed method, a retrospective comparison was conducted among FLAIR images synthesized according to the physics-based model, the hybrid method, and conventional FLAIR images across forty patients.

### Prospective validation on a healthy volunteer

To validate the potential prospective application of the proposed method on patients in the future, a comparison between a prospective and retrospective under-sampling implementation of the hybrid synthetization method was performed on a healthy volunteer with no known history of neurological diseases.

### Quantitative analysis of physics-based, hybrid and conventional FLAIR images from clinical data

The lesion-to-GM ratio, lesion-to-WM ratio, and contrast-to-noise ratio (CNR) were used to compare physics-based, hybrid and conventional FLAIR images. These quantities were computed by first segmenting the lesion and healthy-appearing tissue (grey and white matter) on the FLAIR images.

The lesion-to-GM ratio was calculated as the mean signal intensity of a lesion divided by that of the healthy appearing GM. The lesion-to-GM CNR was calculated as the difference between the mean signal intensity of a lesion and that of the healthy appearing GM divided by the median standard deviation of all tissues in each patient [6]. The median standard deviation of the signal intensity of, respectively, the lesion and healthy-appearing tissue (grey and white matter) of the FLAIR images was defined as the noise [26]. The lesion-to-WM ratio and lesion-to-WM CNR were calculated likewise.

### Statistical analysis

The Wilcoxon signed rank test was used to analyze the difference in the signal ratios and CNRs of physics-based FLAIR images and hybrid FLAIR images. A *p* value smaller than 0.05 was considered as statistical significance.

For five patients, the lesion appeared to be either hypointense (patient ID 36) or composite (hyper and hypointense, patients ID 37–40). See also the yellow arrows in Figure S7. For this reason, we had to exclude these five datasets from the statistical analysis.

## Results

### Retrospective analysis on patients

**Fig. 2:**
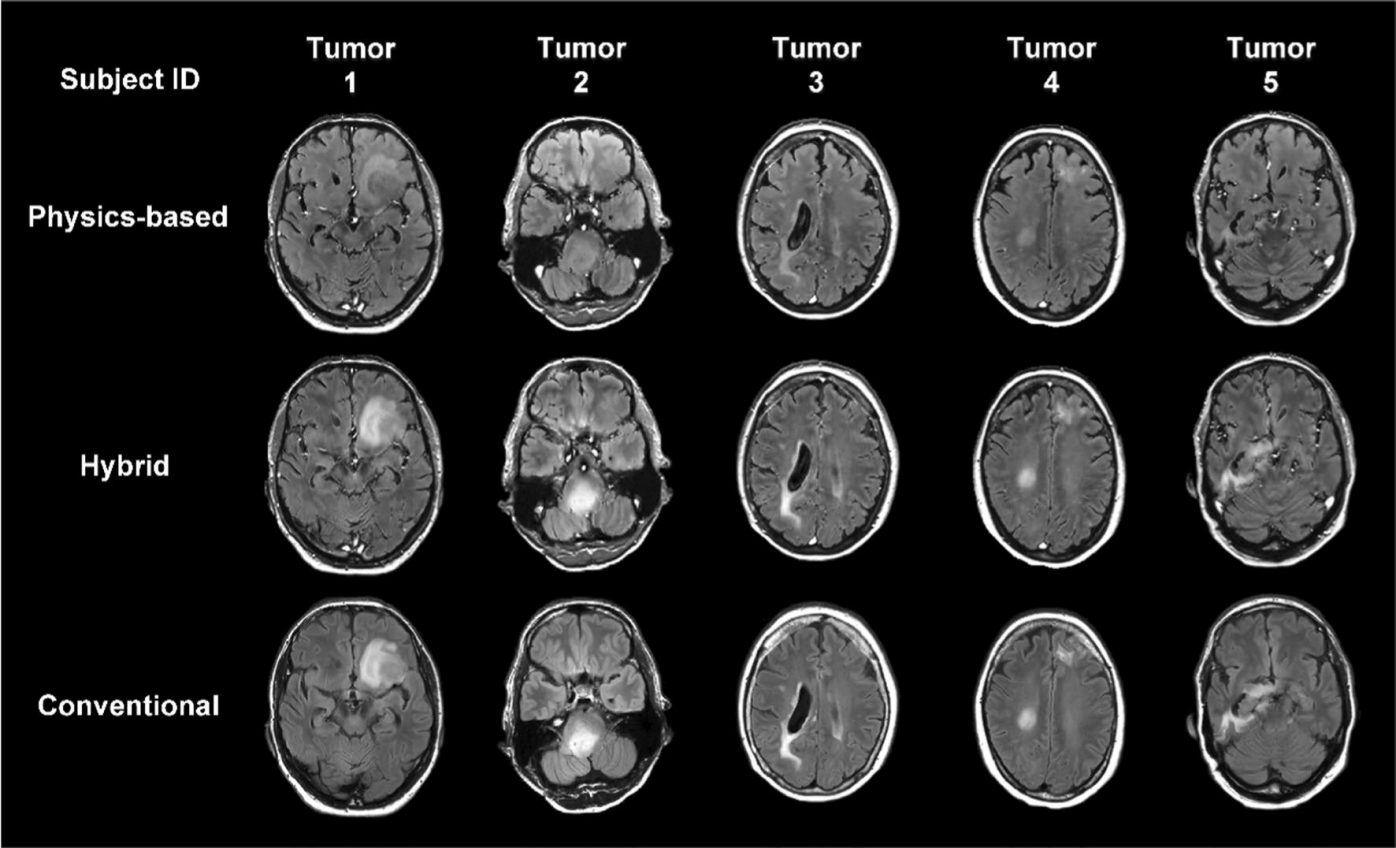
Comparison of FLAIR images synthesized according to the physics-based model, the hybrid method (12.5% of conventional FLAIR acquisition is used) and conventional FLAIR images. The axial slices of interest from the five tumor patients are shown

**Fig. 3:**
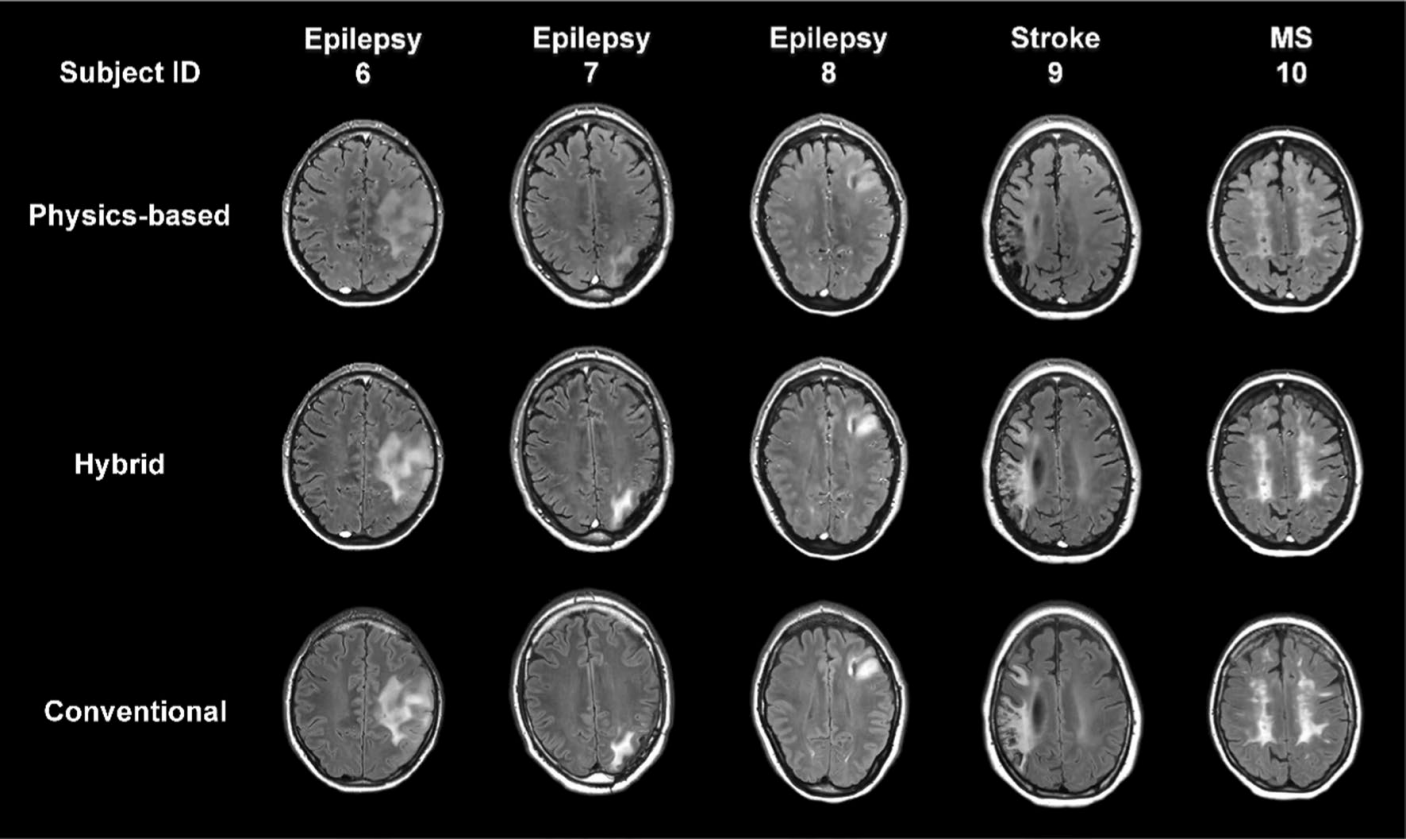
Comparison of FLAIR images as obtained from the physics-based model, the hybrid method and conventional FLAIR. The axial slices of interest from representative patients with (6)-(8) epilepsy, (9) stroke, and (10) multiple sclerosis are shown

The comparison among physics-based synthetization, hybrid synthetization and fully sampled conventional FLAIR images of the 40 patients is shown in Figs. 2–3 and Figures S3-S7. The most representative slice of the 40 patients with tumor, epilepsy, stroke, and multiple sclerosis are displayed. It can be observed that lesions appear hypointense in FLAIR images synthetized with the physics-based model, while in hybrid images and conventionally acquired FLAIR images they appear hyperintense with comparable signal intensity.

**Fig. 4:**
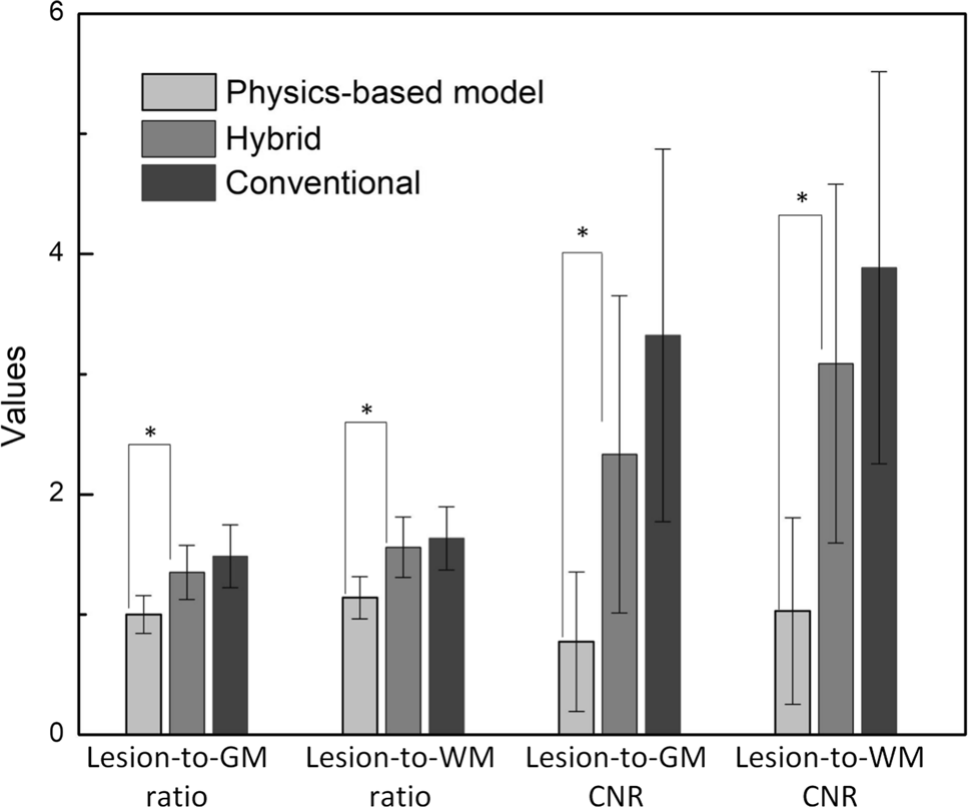
Comparison of signal ratios and contrast to noise ratios among lesions, WM, GM relative to the physics-based model, hybrid method and conventional FLAIR images. All four performance metrics of the hybrid synthetic FLAIR images were significantly higher than those of the physics-based images (Wilcoxon signed rank test: *p < 0.005)

The lesion-to-WM ratio, lesion-to-GM ratio, and their corresponding CNRs relative to the physics-based, hybrid, and conventional FLAIR images are averaged over 35 patients and shown in Fig. 4. It can be observed that all performance metrics of the hybrid FLAIR images were significantly higher than those of FLAIR images from the physical model (Wilcoxon signed-rank test, *p* < 0.005), and comparable to those of conventional FLAIR images.

### Prospective and retrospective analysis on a healthy volunteer

**Fig. 5:**
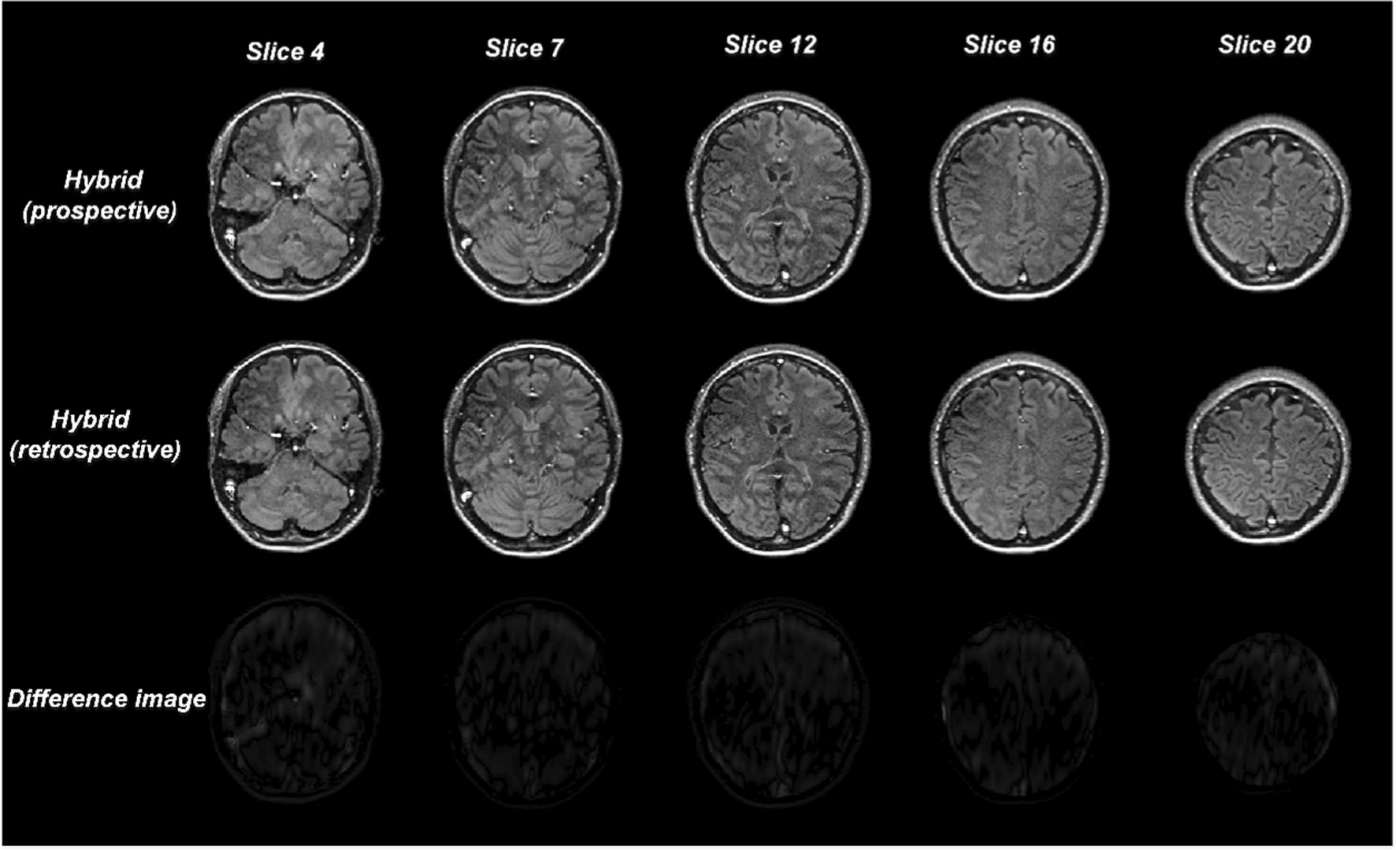
Comparison of prospective and retrospective hybrid FLAIR images from different slices of a healthy volunteer’s brain when applying a 12.5% under sampling in the conventional FLAIR acquisition. The two approaches lead to practically the same result

The comparison between prospective and retrospective FLAIR under-sampling for the hybrid approach is shown in Fig. 5**.** Several slices from one healthy volunteer are shown. The difference between the two reconstructions is also displayed at the bottom of the figure. All images use the same intensity window. The difference between the prospective and retrospective implementation of the hybrid method is negligible, demonstrating the validity of the retrospective analysis of the hybrid method as presented for the patient datasets.

## Discussion

In this study, we demonstrated that the proposed hybrid method can deliver synthetic FLAIR images with improved lesion visualization at the cost of only a very short additional acquisition. The newly proposed hybrid approach to synthetic MRI was compared with conventional FLAIR and the previously adopted physics-based synthetization. Visual inspection as well as quantitative performance metrics showed a higher lesion contrast in hybrid FLAIR images than in physic-based images. This was comparable to the conventional FLAIR images, demonstrating the impactful improvement of such methodology at the cost of only a small increment in acquisition time.

The proposed hybrid method is easy to implement in clinical practice since it requires only the additional acquisition of low-frequency data from conventional FLAIR images to recover important contrast information, Unlike other deep learning methods that rely on large datasets for training [10–13, 27], the hybrid method which is computationally efficient and operationally simple; it also offers greater flexibility when employing different FLAIR sequences compared to data-driven methods that would require retraining for each new sequence [27].

The parameter maps obtained from quantitative MRI can be used to produce synthetic FLAIR images, as well as other contrast weighted images, like *T*1w, *T*2w, and PDw within reduced acquisition time [7]. Furthermore, the quantitative parameters have the potential for providing radiologists with new diagnostic tools for tissue characterization and the ability to monitor disease progression [28–30].

This study has some limitations. Firstly, we only demonstrated a cartesian sampling strategy with a central keyhole-like approach for a single sequence type and focus on improving the lesion appearance. Notably, this technique will likely not enhance image contrast at high spatial frequencies. This limitation will affect the ability to discern changes in FLAIR signal within the subarachnoid space, subdural collections, and smaller white matter lesions. Considering that some degree of blurring is visible in the hybrid FLAIR images as a consequence of the highly under-sampled acquisition, different k-space sampling and merging strategies could be developed, like compressed sensing and prior *cross-contrast* -reconstruction method [13, 31]. Specifically, in compressed sensing, high-frequency information is acquired to preserve structural details and mitigate blurriness. Additionally, from the reconstruction perspective, there are some potential new methods that may address the issue of blurriness. One such method involves prospectively incorporating FLAIR data into the reconstruction of T_1_, T_2_, and PD images. Therefore, a prior-guided joint-reconstruction method can be developed as follows:$\underset{{T}_{1},{T}_{2},PD}{\text{min}}\frac{1}{2}{\Vert {D}_{mrstat}-{f}_{M}({T}_{1},{T}_{2},PD)\Vert }_{F }^{2}+\uplambda {\Vert {D}_{flairkh}-{f}_{p}({T}_{1,}{T}_{2},PD)\Vert }_{F }^{2}$. Where ${D}_{mrstat}$ is the fully sampled MR-STAT measured signal, ${D}_{flairkh}$ is the keyhole measured conventional FLAIR signal, ${f}_{M}$ is the MR-STAT signal model, ${f}_{p}$ is the physics-based model of synthetic FLAIR. This prior joint reconstruction approach forces the reconstructed T_1_, T_2_, PD to (partially) match the undersampled FLAIR data and could thus improve the recovery of synthetic FLAIR, but it also introduces bias in quantitative maps when the regularization term is involved. Another promising extension of the current reconstruction method could be to use a data-driven synthesis model to produce FLAIR from MR-STAT acquisitions and then the heavily undersampled FLAIR data serve as a data-consistency measure to fine-tune and validate synthesis [32]*.* Secondly, we focused on 2D acquisitions since a 3D version of MR-STAT is still in a development phase [33]. Nonetheless, our method could be readily extended to 3D datasets. In fact, we expect that it may offer even better performance in terms of reducing scan time, since the keyhole under sampling strategy could be applied also in the additional phase-encoding direction of 3D FLAIR image. On the other hand, the blurriness issue may be less noticeable, given that 3D keyhole acquisition can cover a larger volume and maintain more structural information. Thirdly, the retrospective nature of this study may also be seen as another limitation, albeit the negligible difference between the hybrid FLAIR prospectively generated on a healthy volunteer demonstrates the validity of the presented approach. Therefore, in the future, a fully prospective validation will also be performed on a larger patient population by acquiring both fully sampled as well as ultra-undersampled conventional FLAIR datasets on 3D acquisitions. Future work will address the validation of the method from a diagnostic perspective by radiological inspection of the new images over a larger patient cohort.

## Conclusion

The proposed hybrid synthetization method improved the lesion appearance in the synthetic FLAIR images by combing a single quantitative MR-STAT acquisition and an ultra-undersampling conventional FLAIR acquisition. This approach results in improved synthetic FLAIR images at the cost of a 13% increase in acquisition time.

## Supplementary Information

Below is the link to the electronic supplementary material.

Supplementary file1 (DOCX 3170 KB)

## Data Availability

The data and code that support the findings of this study are available from the corresponding author upon reasonable request.
