# Supplementary material for: Improving the lesion appearance on FLAIR images synthetized from quantitative MRI: a fast, hybrid approach
Source: MAGMA. 2024 Aug 24;37(6):1021–30. doi: 10.1007/s10334-024-01198-z (PMC11582199; doi:10.1007/s10334-024-01198-z)
Supplement: Supplementary file 1 — Supplementary file1 (DOCX 3170 KB) [file 10334_2024_1198_MOESM1_ESM.docx]

Magnetic Resonance Materials in Physics, Biology and Medicine

*Supplementary material to*

**Improving the lesion appearance on FLAIR images synthetized from quantitative MRI: a fast, hybrid approach**

**Fei Xu^1,2^,** **Stefano Mandija^1,2^,** **Jordi P. D. Kleinloog ^1,2^, Hongyan Liu ^1,2^, Oscar van der Heide ^1,2^,** **Anja G. van der Kolk ^3^, Jan Willem Dankbaar ^4^, Cornelis A.T. van den Berg ^1,2^, and Alessandro Sbrizzi ^1,2^**

*^1^Computational Imaging Group for MR diagnostics & therapy, Center for Image Sciences, University Medical Center Utrecht, Utrecht, Netherlands*

*^2^Department of Radiotherapy, University Medical Center Utrecht, Utrecht, The Netherland*

*^3^Department of Medical Imaging, Radboud University Medical Center, Nijmegen, The Netherlands*

*^4^Department of Radiology, University Medical Center Utrecht, Utrecht, The Netherlands*

Corresponding Author: Fei Xu; Phone number: +31-0659381211; E-mail: [f.xu@umcutrecht.nl](mailto:f.xu@umcutrecht.nl)

In this section, we provide the details to inspect the parameters chosen for combining k-space data (Figure S1) and optimal undersampling factor (Figure S2). Additionally, we provide more patient datasets of the results of retrospective analysis on patients, which aim at testing the robustness of the proposed hybrid method with respect to improve the lesion appearance on FLAIR images. The additional results can be found in Figures S3–S7. We also provide more details to explain the reason we exclude five datasets from the signal ratios and CNRs analysis in Figure S7. However, these five datasets still visually demonstrate the ability of our method to recover lesion contrast compared to physics-based FLAIR.


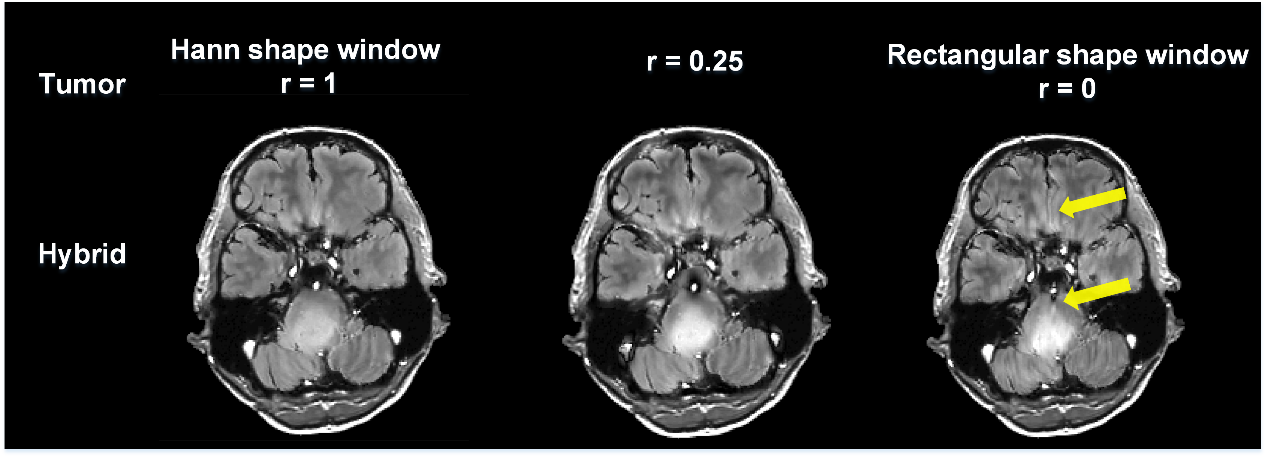


**Fig. S1** The hybrid FLAIR image comparison by using Hann, Tukey and rectangular shape windows. Yellow arrows indicate the truncation artifacts.


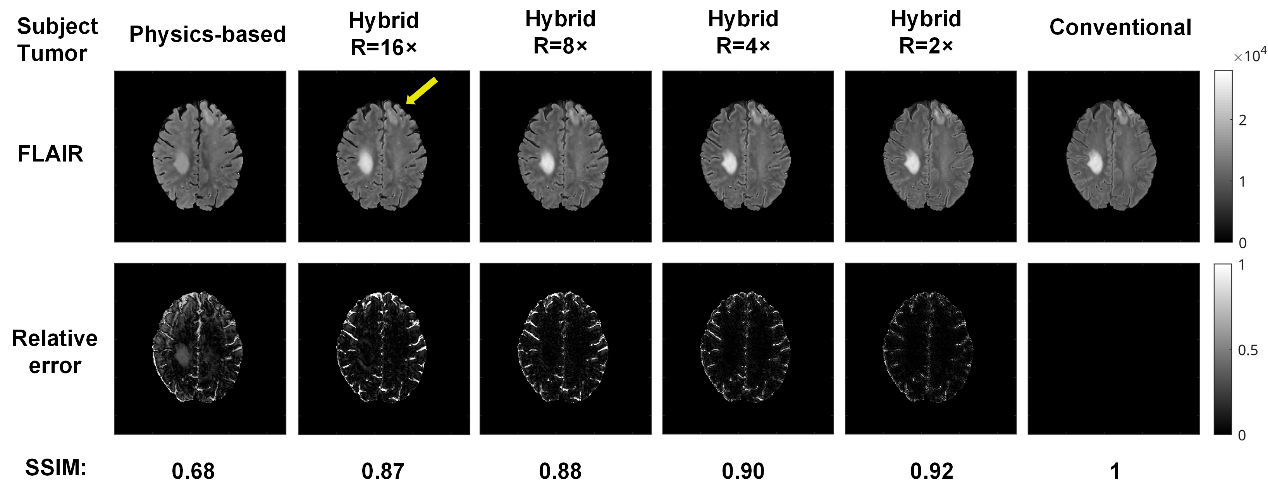


**Fig. S2** Comparison of FLAIR images synthesized according to the physical model, the hybrid method (different accelerating factors of conventional FLAIR acquisition is used) and conventional FLAIR images. The axial slices of interest from one tumor patient are shown.

**
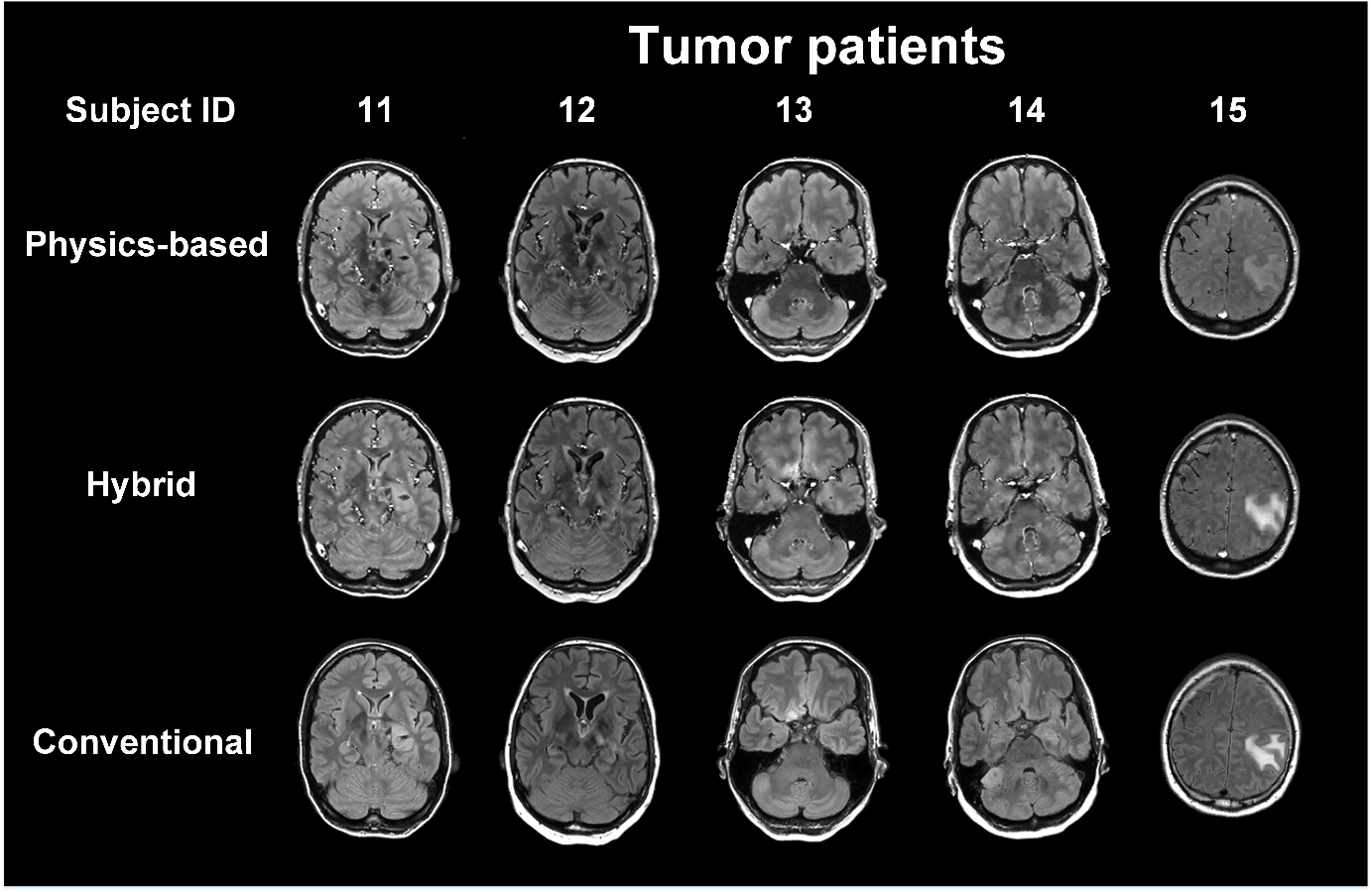
**

**Fig. S3** Comparison of FLAIR images synthesized according to the physical model, the hybrid method and conventional FLAIR images. The axial slices of interest from the five tumor patients are shown.

**
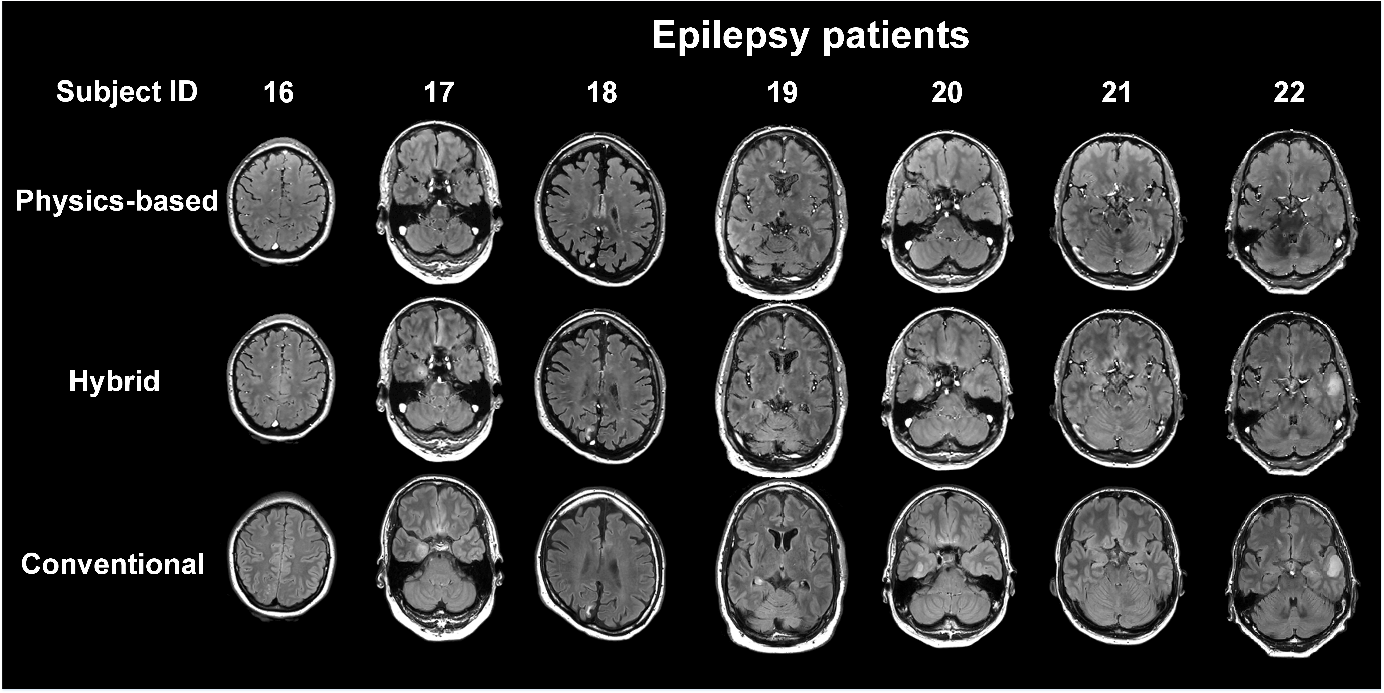
**

**Fig. S4** Comparison of FLAIR images synthesized according to the physical model, the hybrid method and conventional FLAIR images. The axial slices of interest from the seven epilepsy patients are shown.

**
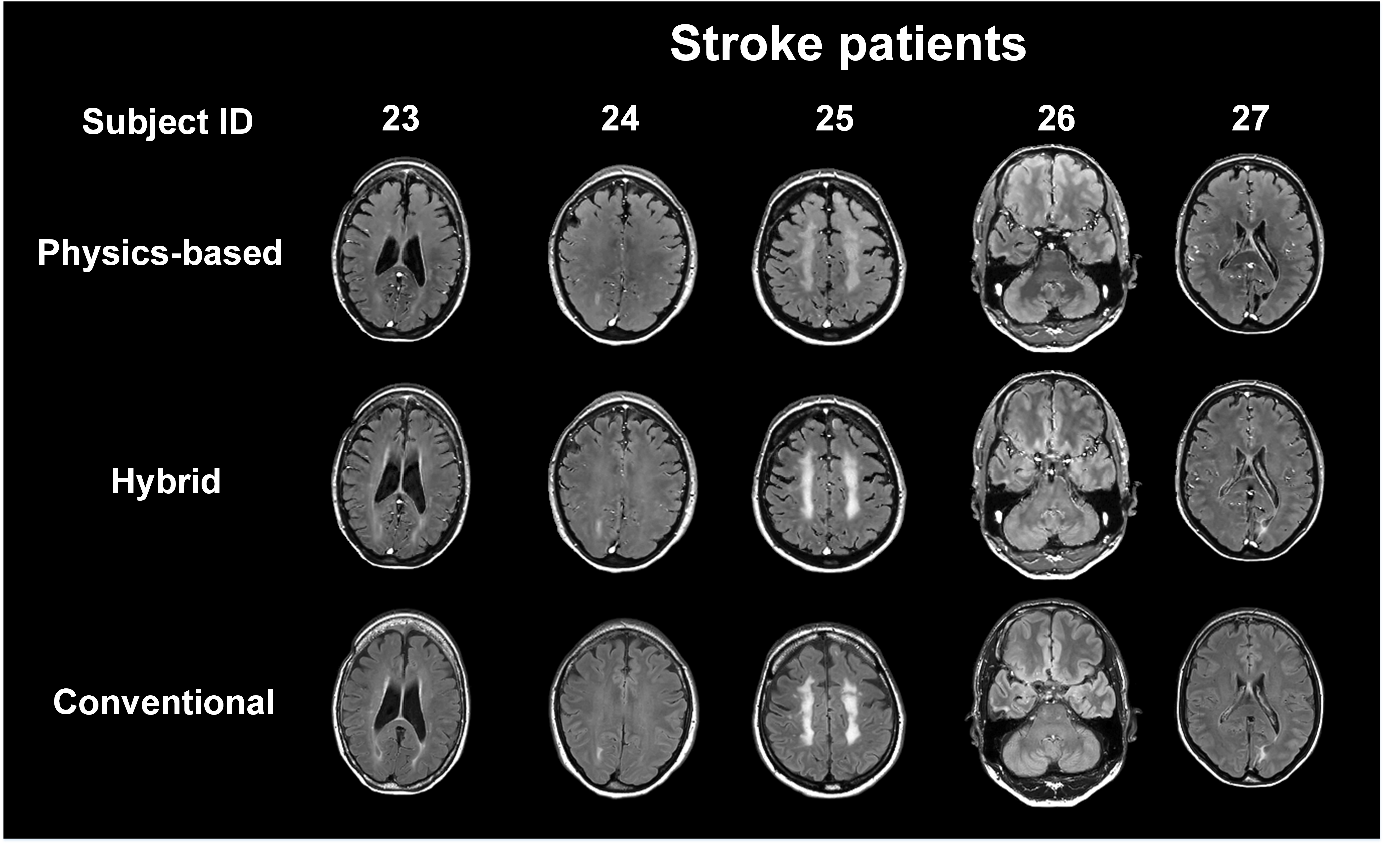
**

**Fig. S5** Comparison of FLAIR images synthesized according to the physical model, the hybrid method and conventional FLAIR images. The axial slices of interest from the five stroke patients are shown.

**
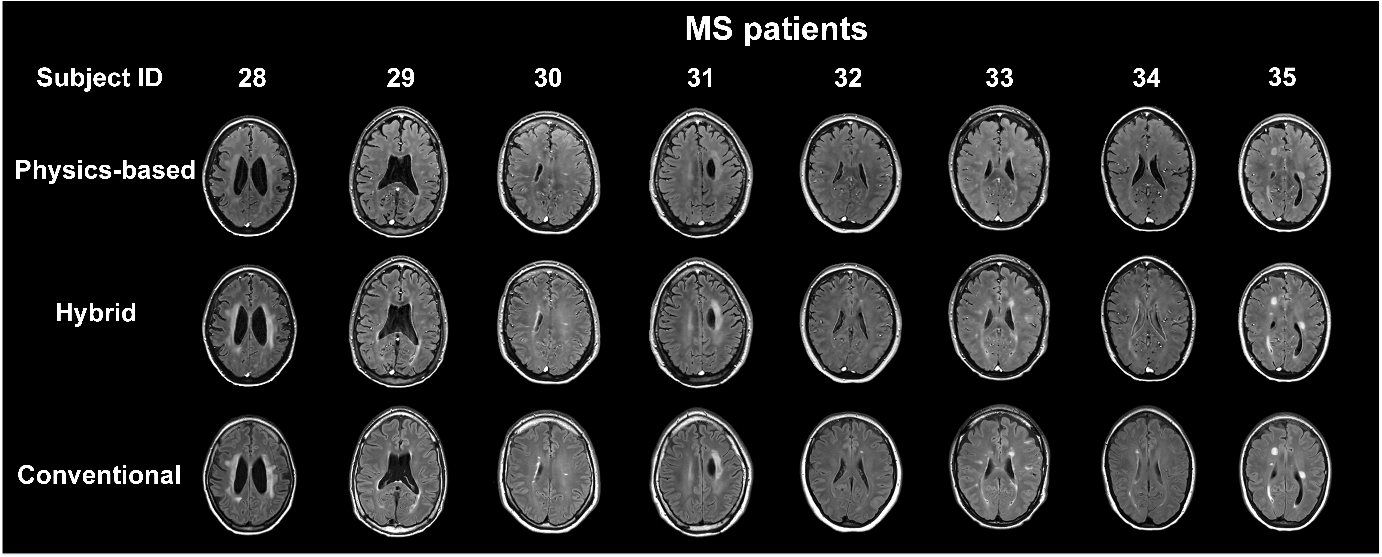
**

**Fig. S6** Comparison of FLAIR images synthesized according to the physical model, the hybrid method and conventional FLAIR images. The axial slices of interest from the eight MS (multiple sclerosis) patients are shown.


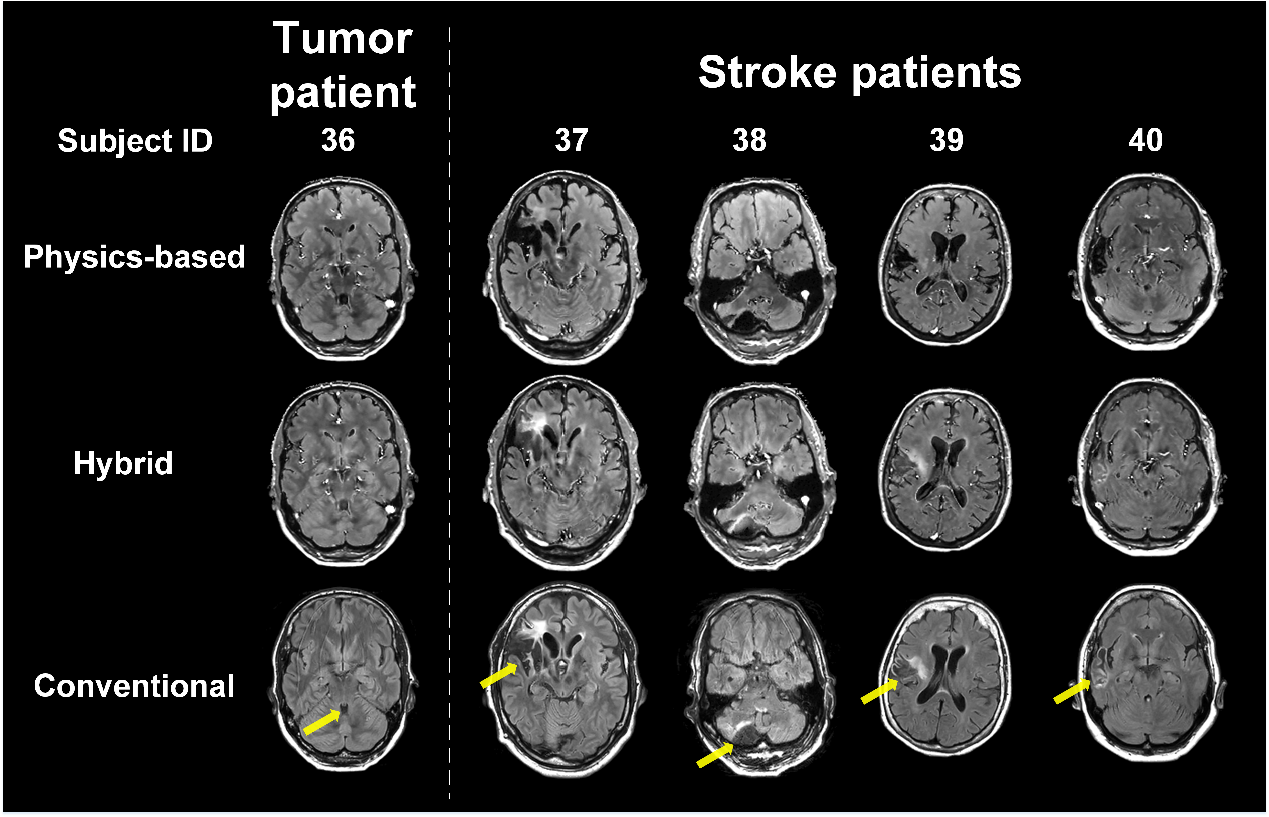


**Fig. S7** Comparison of FLAIR images synthesized according to the physical model, the hybrid method and conventional FLAIR images. The axial slices of interest from the tumor patient and four stroke patients are shown. Arrows indicate the lesion that appeared to be either hypointense (patient ID 36) or composite (hyper and hypointense, patients ID 37-40).
